# Patients with hip fracture and total hip arthroplasty surgery differ in anthropometric, but not cardiovascular screening abnormalities

**DOI:** 10.1186/s12872-020-01792-8

**Published:** 2020-12-02

**Authors:** Regina Csanády-Leitner, Franz J. Seibert, Corinna M. Perchtold-Stefan, Werner Maurer-Ertl, Kathrin Hilgarter, Helmut K. Lackner

**Affiliations:** 1 Division of Physiology, Otto Loewi Research Center, Medical University of Graz, Neue Stiftingtalstraße 6/D05, 8036 Graz, Austria; 2 Department of Orthopaedics and Trauma, Medical University of Graz, Auenbruggerplatz 5, 8036 Graz, Austria; 3 Department of Psychology, University of Graz, Universitätsplatz 2/DG, 8010 Graz, Austria

**Keywords:** Hip fracture, Total hip arthroplasty, Cardiovascular reactivity, Heart rate variability, Aging

## Abstract

**Background:**

With the rising number of hip surgeries, simple and cost-effective tools for surgery risk assessment are warranted. The analysis of heart rate variability (HRV) may not only provide critical insights into the general frailty of patients with hip surgery, but also allow for better differentiation of health profiles in different hip surgery groups. Using HRV analysis, the present study compared cardiovascular as well as anthropometric parameters between patients with hip surgery, the hip fracture surgery group (HFS) and the total hip arthroplasty group (THA), and a control group.

**Methods:**

71 participants (56.3% women), aged 60–85 years, took part, divided into three groups—patients after hip surgery (21 HFS and 30 THA patients) and a control group (20 participants). Electrocardiogram was recorded at baseline and after the application of a physical stressor (grip strength). A 3 (group) × 2 (time) repeated measures ANOVA, and a chi square test were carried out to test for group differences.

**Results:**

Higher weight (*p* = .002), body mass index (*p* = .001), and systolic blood pressure (*p* = .034) were found in THA patients compared to HFS patients. Lower calf circumference (*p* = .009) and diastolic blood pressure (*p* = .048) were observed for the HFS group compared to the control group. For cardiovascular parameters, significant differences emerged between the HFS group and the control group in HR (*p* = .005), SDNN (*p* = .034) and SD2 (*p* = .012). No significant differences in cardiovascular parameters were observed between the two hip surgery groups: neither at baseline nor during stressor recovery.

**Conclusions:**

While HRV seems to differentiate well between HFS patients and controls, more research with larger samples is needed to scrutinize similaritites and differences in cardiovascular profiles between HFS and THA patients.

## Background

Hip fractures have been recognized as an established global health problem due to their pronounced posteroperative complications, including chronic pain, disability, diminished quality of life, and premature death [1]. Klicken oder tippen Sie hier, um Text einzugeben. Moreover, incidences of both hip fracture surgeries and total hip arthoplasties are rapidly increasing along with the number of elderly patients, due to demographic changes and rising life expectancy [2–4]. Since most fracture patients are above 65 years old, tend to be frail with multiple comorbidities, and tend to have experienced indoor or outdoor falls that may have entailed permanent disability [5–9], minimizing surgery complications is vital.

One way to conduct simple, non-invasive, and cost-efficient risk assessment is by analysing heart rate variability (HRV). Providing critical information on the functioning of the autonomic nervous system, HRV has proven to be useful in predicting mortality and helping to identify patients in need of increased surveillance or prophylactic treatment [10]. HRV, which denotes the fluctuation in time intervals between adjacent heartbeats, is often used in verifying the health status of individuals after an intervention [11, 12]. Stressful tasks, as observed in several studies, lead to heart rate (HR) acceleration, blood pressure increase and enhanced respiratory frequency, but also to a decrease in HRV [13]. Thus, HRV delivers information on the effect of stress, and is a suitable for measuring the effect of cardiovascular stress, and hence, cardiovascular risk stratification; this is often evaluated by linear and nonlinear signal analysis methods [14, 15].

In light of the well documented benefits of HRV measurement for monitoring pre, peri and postoperative instability in order to minimize major cardiac events and mortality [11–13, 16, 17], the present study aimed to extend previous findings by comparing different HRV parameters between three groups of participants: patients after hip fracture surgery (HFS), patients after total hip arthroplasty surgery (THA), and a control group. HRV parameters were compared both before and after the application of a physical stressor (a grip strength test, used as a riable proxy for whole body strength) [18]. These comparisons were supplemented by anthropometric parameters, in order to gain a more comprehensive insight into the general frailty of patients post-surgery. Importantly, comparing HRV parameters for two distinct types of hip surgeries (HFS, THA) may allow for a better differentiation of health profiles of respective patient groups, and help match patients to treatments targeting their individual deficits.

Based on their respective symptoms and presumed greater frailty, we hypothesized that HFS patients would show significantly poorer HRV and anthropometric results compared to the THA and the control group.

## Methods

### Setting and participants

This study was conducted at the Medical University of Graz Department of Orthopaedics and Trauma. A total number of 189 people were asked between October 2016 and June 2017 to participate in the study. Participants were defined either as patients with traumatic hip fracture, followed by a hip fracture surgery (HFS), or total hip arthroplasty (THA) admitted to surgery, and people without a current surgery, which comprised the control group. The study was performed according to the convention of the Declaration of Helsinki, 1964 and approved by the local ethics committee (EK-28-515 ex 15/16). Written informed consent prior to the investigation was obtained from all participants, after they had received detailed information about the investigation and the study protocol.

### Baseline characteristics

Eligibility criteria consisted of the following: age between 60 and 85 years, German language speaking (to understand the procedure instructions), body mass index (BMI) below 35, and a current hip surgery (except for those in the control group). We excluded patients who had dementia or other serious cognitive impairments, neurodegenerative diseases such as Parkinson´s disease, autoimmune and musculoskeletal disorders such as multiple sclerosis, severe visual and auditory impairments, as well as those with beta-blocker intake or abuse of alcohol or drugs. Since elderly individuals are likely to experience an imbalance in autonomic nervous activity due to surgical stress, we decided to start the testing after the third day post-surgery. The testing was carried out between the third and tenth day after surgery, and circadian rhythms were taken into account [11, 19].

### Testing conditions

Anthropometric measurements were conducted using a calibrated electronic scale (Seca Modell 799, Germany) to measure weight (participants were asked to remove shoes), and body height measured to the nearest 0.1 cm with an anthropometer (GPM 100, Rudolf Martin Antropometer, Switzerland). Calf circumference (CC) was taken using a tape measure. Measurements were performed according to the International Society for the Advancement of Kinanthropometry (ISAK) protocol [20]. The testing time (between 3 and 7 PM) was kept constant, following circadian rhythms; room temperature was also kept constant and the laboratory was kept quiet to exclude any interrupting noise. Participants were required to abstain from caffeine, alcohol and heavy meals for two hours prior to testing. After familiarizing participants with the experimental protocol, disposable ECG electrodes were attached at three points on the participant´s chest. During the entire experimental procedure, patients had to sit quietly in a comfortable chair that was adjusted for each person, without speaking or moving abruptly. Our protocol consisted of an adaptation period followed by a 3 min measurement at rest, to record baseline results; this was measurement time point (MTP) 1. Thereafter, participants were instructed to press a grip strength dynamometer as strongly as possible for three seconds, twelve times with twelve seconds break after each turn, to produce physical stress. Finally, a further 3 min measurement at rest followed, to record recovery results (MTP 2); this concluded the investigation. Prior to and also following ECG recordings, blood pressure was measured using a standardized hospital device (Boso Clinicus I, Germany).

### Data acquisition and preprocessing

Continuous ECG was recorded using the exercise physiology and software system Powerlab 8/35, with LabChart Pro Software all from ADInstruments (Sydney, Australia), with a sampling rate of 1000 Hz. Grip strength, derived from a grip strength transducer, ECG and respiration frequency, derived from a chest-strap, were indicated by three channels on the device. Disposable electrodes were fixed at the thorax (2-lead, 1 channel position), and bipolar limb derivation using an Eindhoven Lead II set-up was chosen. The recorded binary data were saved as European Data Format, EDF [21]. The R-wave detection was carried out using a revised MATLAB-function (MATLAB®, Mathworks Natick, Massachusetts, USA) and the immediate beat to beat heart rate was also calculated using this function [21]. Artifact handling was performed semi-automatically by a visible check of every signal, in combination with a Matlab-function which identified these signals according to the following criteria: (1) ectopic beats, (2) physiological limits and (3) maximal percentage of change in relationship to standard deviation of the signal. Therefore, we used time series with equidistant time steps, after resampling beat to beat values at 4 Hz, using piecewise cubic spline interpolation. Single artifacts were replaced by linear interpolation and only time series with 85% validity were accepted.

The signal measurement and the subsequent processing were performed according to international recommendations of *linear* (time domain, TD: the changing of signals over time, and frequency domain, FD: the frequency of signals in a special range) and *nonlinear* HRV parameters [22].

In regards to linear parameters, *Time domain* variables of HRV were generated using the standard deviation of normal to normal R-R intervals, *SDNN,* in *ms* (R is the peak of a QRS complex—heartbeat, HR), which is responsible for the variations in heart beat, to reflect sympathetic and extreme vagal tone. The root mean square of successive heartbeat interval differences, *RMSSD,* in *ms*, correlates with the frequency domain variable ´high frequency´, *HF,* listed below*,* and estimates variations in the HR. *Frequency domain* variables, using the power spectral density, were differentiated into low frequency, *LF,* in *ms*^*2*^*,* defined by the power of the “low”-frequency band 0.04–0.15 Hz, and *HF,* in *ms*^*2*^*,* the power of the “high”-frequency band 0.15–0.4 Hz. Due to skewed distributions of frequency domain indexes, a natural logarithm, ln, transformation was applied; *LnLF* is related to mainly sympathetic factors, *lnHF* is related to the vagal influence, and *lnLF/HF* is considered to reflect the sympathovagal balance.

Nonlinear parameters, describing the SD of an average variability around a mean, are expressed in the standard deviation of the short-term NN, normal to normal R-R intervals, interval variability, *SD1,* in *ms,* the standard deviation of the long-term NN interval variability, *SD2,* in *ms,* and the ratio between *SD2* and *SD1*, *SD2/SD1.*

### Statistical analyses

Data are presented as means, M, ± standard deviation, SD, as well as minimum and maximum values. Normal distribution was checked by normal probability plots and homogeneity of variance was checked by Levene’s test. *LF* and *HF* were log transformed to achieve normal distribution. Group differences at baseline and recovery were investigated using ANOVAs (Analysis of Variance). The differences in change of groups over time was evaluated by a 3 (group: HFS, THA, control; between-subjects factor) × 2 (time: baseline, recovery; within-subjects factor) repeated measures ANOVA. The chi square test was chosen to validate group affiliations and gender distribution. A probability of *p* < 0.05 was considered to be significant. All statistical analyses were performed using the Statistical Packages for the Social Sciences, SPSS, IBM SPSS Statistics Version 24 for Windows (SPSS Inc., Chicago, USA).

## Results

### Study participants

**Fig. 1:**
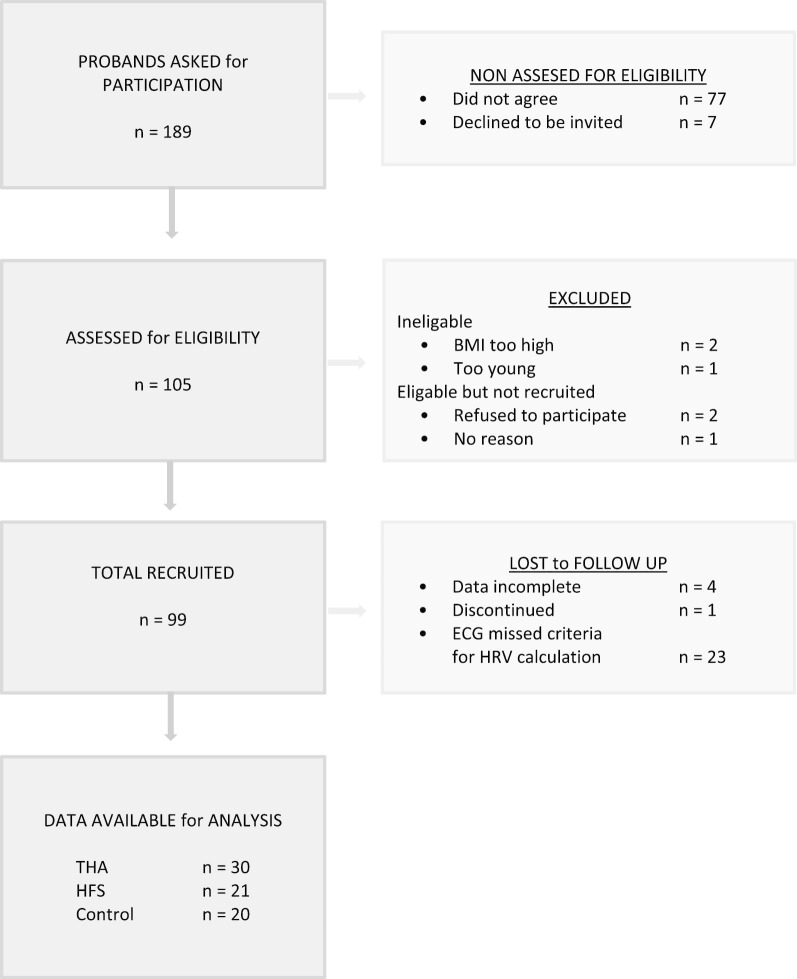
Flow diagram showing the recruitment of participants. n denotes number, BMI body mass index, THA total hip arthroplasty, HFS hip fracture surgery, ECG electrocardiogram and HRV heart rate variability

Out of 189 possible participants, 99 (52.3%) agreed to take part in the study. Of these, 23 had to be excluded due to not meeting the electrocardiogram (ECG) entry requirements in regards to pathological reasons, and another 5 patients were excluded due to incomplete data or not continuing with the study, a total of 28.3%; see Fig. 1. The operated groups showed a significantly higher dropping out rate, χ^2^ (1) = 4.4845, *p* = 0.034, ϕ = 0.34 than the control group (in the HFS group 44.7%, THA group 25% and the control group 4.8%). Finally, 71 participants (31 male and 40 female, the number of women in the HFS group was higher, compared to the other groups), 51 of which underwent surgery, were eligible for analysis; the THA group comprised of 30 participants, the HFS group 21, and 20 in the control group.

### Anthropometric parameters.

Age was not significantly different between the groups. The mean age of the THA group was 71 ± 6.7 years, the HFS group 74 ± 5.5 years and the control group 71 ± 6.7 years, as presented in Table 1.

**Table 1:** Anthropometric characteristics of the study groups

	THA n = 30	HFS n = 21	Control group n = 20	*p* value
Male/female	(17/13) Mean (± SD) min/max	(6/15) Mean (± SD) min/max	(8/12) Mean (± SD) min/max	
Age [year]	71 (± 6.71) 61/85	74 (± 5.5) 62/84	71 (± 6.72) 60/83	.105
Height [cm]	170.5 (± 8.95) 151/187	167.2 (± 9.10) 154/180	169.4 (± 8.40) 158/187	.477
Weight, [kg[	76.9 (± 9.72) 60/100	65.1 (± 12.52) 44/95	72.7 (± 13.36) 52/103	.003*
BMI [kg/m^2^]	26.5 (± 2.84) 21/33	23.1 (± 3.64) 17/33	25.2 (± 3.23) 21/34	.002*
Calf circumference [cm]	35.6 (± 3.60) 29/43	33.9 (± 3.30) 29/41	37.1 (± 2.37) 31/41	.012*
SBP [mmHg]	132 (± 13.0) 105/160	123 (± 10.01 100/140	129 (± 11.16) 99/145	.038*
DBP [mmHg]	76 (± 8.36) 55/90	71 (± 7.82) 55/80	77 (± 7.61) 65/90	.026*
Grip strength [N]	191.8 (± 70.3) 82.4/306.8	152.4 (± 103.3) 38.4/438.5	213.5 (± 78.4) 90.4/345.9	.022*
Grip strength z-transformed by gender	− 0.04 (± 0.856) -1.97/1.34	− 0.31 (± 1.191) − 1.55/− 0.31	0.38 (± 0.869) − 1.81/1.87	.080

Anthropometric data of the study groups are shown as means (± SD) and minimums and maximums of age_[y]_, height_[cm]_, weight_[kg]_, BMI_[kg/m_^2^_]_, calf circumference_[cm]_, SBP_[mmHg]_, DBP_[mmHg]_, grip strength_[N]_ and grip strength transformed by gender. Significant results of the analysis of variance are presented and marked by an asterisk (*)

*SD* standard deviation, *THA* total hip arthroplasty, *HFS* hip fracture surgery, *BMI* body mass index, *SBP* systolic blood pressure, *DBP* diastolic blood pressure, *[y]* year, *[cm]* centimeter, *[kg]* kilogram, *[m*^*2*^*]* square meter, *[mmHG]* millimeters of mercury, *[N]* Newton, *[p]* significance, *p < .05

**Fig. 2:**
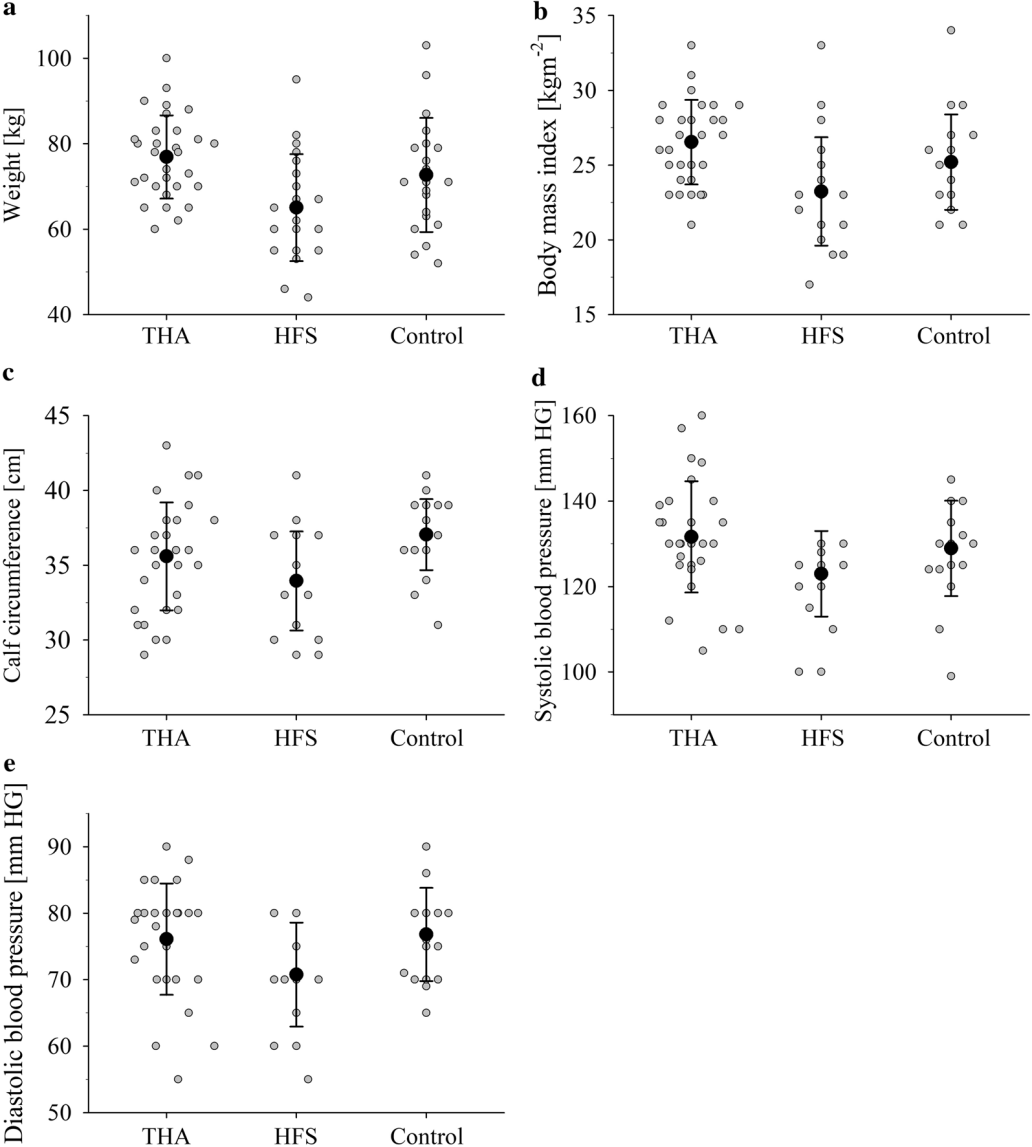
Anthropometric parameters are presented for significant differences between the groups (as observed in post-hoc tests). Weight (**a**), BMI (**b**), CC (**c**), systolic (**d**) and diastolic (**e**) blood pressure. Post hoc tests showed significant differences in weight, BMI and systolic blood pressure between THA and HFS patients, whereas CC and diastolic blood pressure differed significantly between HFS patients and the control group. THA describes total hip arthroplasty, HFS hip fracture surgery, BMI body mass index, CC calf circumference and *p* is *p* value

**Fig. 3:**
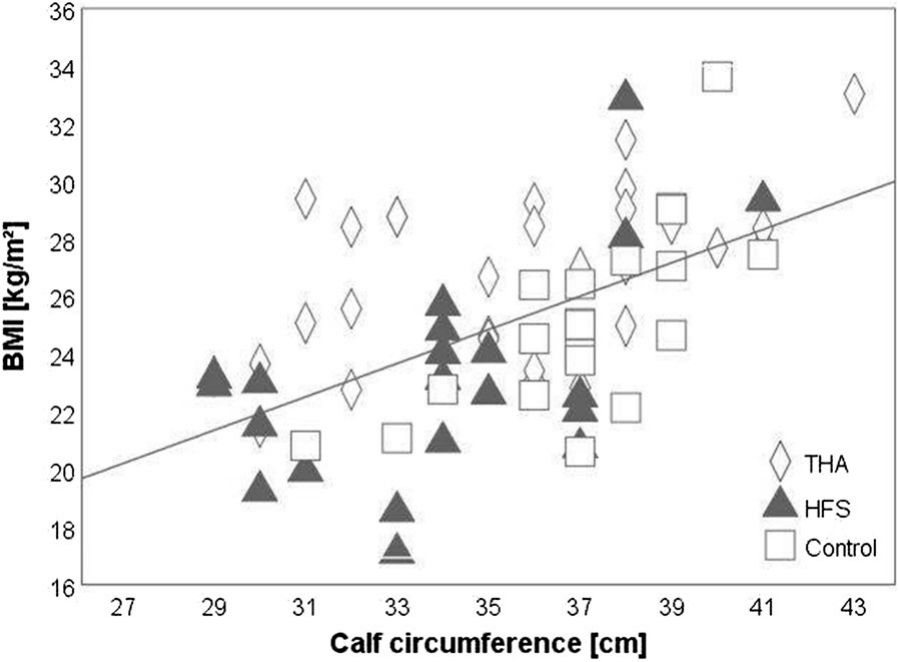
Correlations are presented between BMI and CC for THA, HFS and control groups. Relationship between body mass index (BMI) and calf circumference (CC) is mapped. The BMI (kg/m^2^) is compared with CC (cm) as an indicator for sarcopenia. There was a strong positive correlation (r = .566, *p* < .001, marked by a line) between BMI and CC among the groups, THA (30), HFS (21) and Control (20) groups. Eleven people: 5 THA (3 male, 2 female), 1 HFS (male) and 5 in the Control group (3 male, 2 female), 15% in total, had elevated CC. Above the set limit (> 38 cm), an increase of fall risk can be observed and mobility disability is more likely [30]. THA denotes total hip arthroplasty, HFS hip fracture surgery, BMI body mass index, kg kilogram, cm centimetre and m^2^ means square meter

The following parameters showed significant differences between the three groups: BMI (*p* = 0.002) weight (*p* = 0.003), CC (*p* = 0.012), systolic blood pressure (SBP) (*p* = 0.038) and diastolic blood pressure (DBP) (*p* = 0.026), as seen in Table 1. Post hoc comparisons showed significant differences between the HFS group and the other groups: Higher weight (*p* = 0.002), BMI (*p* = 0.001), and systolic blood pressure (*p* = 0.034) were observed in the THA compared to the HFS group, whereas lower CC (*p* = 0.009) and diastolic blood pressure (*p* = 0.048) were observed in the HFS compared to the control group, as presented in Fig. 2. A strong positive correlation (r = 0.566, *p* < 0.001) between BMI and CC among the groups was observed. Six out of 71 participants (8.5%) had a combination of BMI which was either normal or too low, and a CC which was too low, in accordance with recommended levels. Eleven (5 THA and 1 HFS) participants (15%) had elevated CC (> 38) combined with elevated BMI (> 25), seen in Fig. 3.

### Grip strength testing

In the analysis of grip strength, significant differences were observed between the three groups (*p* = 0.022). Post hoc tests showed significantly lower results for the HFS group compared to the control group. To minimize the influence of gender, data were z-transformed, rendering the group comparisons significant at only trend level (*p* = 0.080). Further results are shown in Table 1.

### HRV parameters

An overall illustration of cardiovascular parameters is presented in terms of linear and non-linear HRV indices, divided according to the three study groups, in Table 2. Results for the 3 (group) × 2 (time) repeated-measures ANOVA are shown in Table 3.

**Table 2:** Descriptive statistics of linear and non linear HRV indices at baseline and recovery among the study groups

		Baseline Mean(± SD)			Recovery Mean(± SD)		
MA	Male/female	THA n = 30 17/13 Mean (± SD)	HFS n = 21 6/15 Mean (± SD)	Control n = 20 8/12 Mean (± SD)	THA n = 30 17/13 Mean (± SD)	HFS n = 21 6/15 Mean (± SD)	Control n = 20 8/12 Mean (± SD)
TD	HR [bpm]	78.14 (± 10.76)	82.49 (± 12.44)	71.42 (± 11.63)	78.99 (± 11.24)	83.19 (± 12.56)	70.88 (± 11.49)
	SDNN [ms]	19.08 (± 12.19)	14.58 (± 6.95)	24.15 (± 17.83)	22.49 (± 11.65)	18.67 (± 8.30)	28.58 (± 16.31)
	RMSSD [ms]	14.37 (± 19.46)	10.05 (± 7.05)	18.62 (± 19.51)	14.40 (± 19.53)	12.67 (± 11.01)	16.97 (± 16.58)
	RR [breath/min]	17.35 (± 4.07)	17.13 (± 4.08)	16.6 (± 3.90)	18.23 (± 4.55)	18.26 (± 4.42)	16.65 (± 3.74)
FD	ln LF [ms^2^]	3.97 (± 1.21)	3.35 (± 1.07)	4.48 (± 1.23)	4.14 (± 1.28)	3.85 (± 1.04)	4.67 (± 1.06)
	ln HF [ms^2^]	3.31 (± 1.29)	2.78 (± 1.30)	3.89 (± 1.54)	3.23 (± 1.38)	2.98 (± 1.50)	3.81 (± 1.27)
	LF/HF [-]	0.66 (± 1.12)	0.57 (± 0.96)	0.58 (± 0.92)	0.96 (± 1.02)	0.86 (± 1.22)	0.85 (± 0.66)
PPA	SD1 [ms]	10.18 (± 13.80)	7.12 (± 5.00)	13.19 (± 13.83)	10.20 (± 13.84)	8.98 (± 7.80)	12.03 (± 11.76)
	SD2 [ms]	23.93 (± 12.36)	19.02 (± 9.06)	30.83 (± 21.62)	28.89 (± 11.94)	24.23 (± 10.13)	38.15 (± 20.48)
	SD2/SD1 [%]	3.28 (± 1.39)	3.33 (± 1.52)	3.14 (± 1.58)	4.22 (± 2.24)	4.03 (± 2.32)	4.05 (± 1.68)

Baseline is before and recovery after grip strength task, MTP measurement time point, SD standard deviation, HRV heart rate variability, THA total hip arthroplasty, HFS hip fracture surgery, MA method of analysis, TD time domain, FD frequency domain, PPA Poincaré-Plot analysis, HR [bpm] heart rate mean in beats per minute, SDNN the mean of the time of two successive heart beats, ms millisecond, RMSSD root mean square of successive differences, RR respiratory rate, ln natural logarithm, LF low frequency, HF high frequency, LF/HF ratio of low frequency and high frequency, SD1 standard deviation of the short-term NN interval variability, SD2 standard deviation of the long-term NN interval variability, SD2/SD1 ratio between SD2 and SD1

**Table 3:** Results for the 3 (group) × 2 (time) repeated measures ANOVA presented as p-values for the three study groups

Cardiovascular parameters	HR	SDNN	RMSSD	RR	ln LF	ln HF	LF/HF	SD1	SD2	SD2/SD1
Main effect time	.160	< .001*	.596	.008*	.002*	.872	.002*	.596	< .001*	< .001*
Main effect group	.007*	.043*	.463	.631	.024*	.070	.964	.463	.015*	.945
Interaction effect time*group	.042*	.861	.034*	.201	.226	.356	.967	.034*	.659	.862

The main effects of time and then group are presented individually, followed by the interaction effect time*group which presents the effect of time depending on the group. Significant effects are highlighted with an asterisk (*)

THA total hip arthroplasty, HFS hip fracture surgery, HR (bpm) heart rate mean in beats per minute, SDNN the mean of the time of two successive heart beats, ms millisecond, RMSSD root mean square of successive differences, RR respiratory rate, ln natural logarithm, LF low frequency, HF high frequency, LF/HF ratio of low frequency and high frequency, SD1 standard deviation of the short-term NN interval variability, SD2 standard deviation of the long-term NN interval variability, SD2/SD1 ratio between SD2 and SD1, * significant *p* < .05, *p* level of significance

**Fig. 4:**
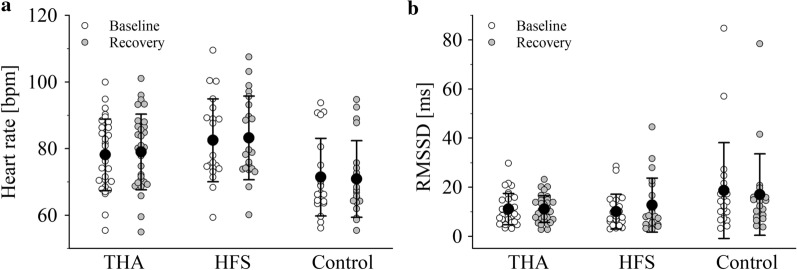
Cardiovascular activity triggered by grip strength task. Analysis of variance (ANOVA) showed differences in changes in the groups over time: significant interactions were found in HR (**a**), F(2,68) = 3.317 *p* = .042 and RMSSD (**b**), F(2,68) = 3.562 *p* = .034. Values are shown as mean value ± standard deviation. THA denotes total hip arthroplasty, HFS hip fracture surgery, HR (bpm) heart rate in beats per minute, RMSSD (ms) root mean square of successive differences in milliseconds, F F-value and *p* is *p* value

At baseline, significant group differences were found in *HR* (*p* = 0.011), *lnLF* (*p* = 0.012), *lnHF* (*p* = 0.040) and *SD2* (*p* = 0.044). At recovery, significant group differences were seen in *HR* (*p* = 0.005), *SDNN* (*p* = 0.041) and *SD2* (*p* = 0.010). Post hoc tests showed significant differences between the HFS group and the control group at baseline, indicating higher *HR* (*p* = 0.009), but lower *lnLF* (*p* = 0.009), lower *lnHF* (*p* = 0.034) and lower *SD2* (*p* = 0.009) for the HFS group. At recovery, the HFS group demonstrated higher *HR* (*p* = 0.004), but lower *SDNN* (*p* = 0.037) and lower *SD2* (*p* = 0.009) compared to the control group. No significant differences in any cardiovascular parameters were observed between the HFS and the THA group (all *p*’s > 0.060). Differences in the main effect time were seen in *SDNN* (*p* = < 0.001), *RR* (*p* = 0.008), *lnLF* (*p* = 0.002), *LF/HF* (*p* = 0.002), *SD2* (*p* = < 0.001) and *SD2/SD1* (*p* = < 0.001). Significant differences between the groups, main effect group, were seen in *HR* (*p* = 0.007), *SDNN* (*p* = 0.043), *lnLF* (*p* = 0.024) and *SD2* (*p* = 0.015). The interaction effect time*group was significant in *HR* (*p* = 0.042) and *RMSSD* (*p* = 0.034), as shown in Fig. 4.

## Discussion

This study compares anthropometric and HRV parameters among participants with and without hip surgery. For HRV parameters, significant differences were observed between patients after hip surgery (HFS and THA groups) and the control group (both at baseline and during stressor recovery). However—and this was surprising and unexpected – no significant HRV differences emerged between the HFS and THA groups.

### Anthropometric parameters

A well-functioning physical condition is made up of a collaboration of many factors. Anthropometric parameters such as weight, BMI, CC, SBP and DBP, included as part of this investigation, can provide information on the physical state of a person.

A significantly higher CC, observed in the control group relative to the HFS group, led to the assumption of higher physical activity and a more active lifestyle for those in this group. This is backed by other research: Kawakami et al. described a relationship between a higher CC and a more active lifestyle with higher levels of physical activity [23]. Dargant-Molina et al. demonstrated a connection between hip fracture and reduced CC, which was also in line with our findings [24]. A decreased CC, observed in the current study in the HFS and THA groups compared to the control group, could possibly indicate a stage of sarcopenia, which is related to a more inactive lifestyle; this appears particularly relevant for those in the HFS group. This connection has been described by Kim and colleagues, who reported on the relationship of CC and sarcopenia, in relation to an inactive lifestyle [23, 25]. Further, the correlation between BMI and CC in the current study showed that 8.5% of study participants could be classified as having a normal BMI but a lower CC, than is recommended. A beginning sarcopenia might be a possibility among these participants. Pérez-Zepedaa and Gutiérrez-Robledob have reported on a possible mobility disability and fall risk for persons with elevated CC (> 38) [26]. In our investigation, six participants in the THA group and one participant in the HFS group had elevated CC but no detailed diagnosis and evaluation concerning mobility disability was done. A more precise diagnosis of these patients should be carried out in general.

Grip strength was used as a reliable proxy for whole body strength, an effect documented by Di Monaco, and showed a trend to significance between the controls and the hip operated participants (*p* = 0.08) [18]. Di Monaco also highlighted the predictive information of grip strength for the overall physical health of a person. In the current study, those in the control group pressed the grip strength dynamometer most strongly, indicating greater physical strength and presumably a more active lifestyle (in line with the results of lower CC and BMI in the operated patients), compared to the operated groups. Our results therefore correspond with those of Di Monaco and colleagues, regarding this strength effect as an important marker for physical health. The control group supported these findings, pressing the grip strength dynamometer the strongest, perhaps indicating a more active lifestyle and greater physical strength, compared to the operated groups.

### HRV parameters

The results of *HR,* elevated at MTP 2 in the operated groups, are in accordance with findings from Goldberger and colleagues, who also found elevated values after a challenge [27]. A decline of HR at recovery compared to baseline was seen in the control group; this may be due to excitement before the trial which may have contributed to an elevated HR at MTP 1. A normal reduction of HR after the challenge was observed in the study groups. However, the expected differences in HR between the operated groups (HFS and THA) could not be confirmed in this investigation.

*RMSSD* differed significantly among the study groups over time. At MTP 1, the lowest values of RMSSD were observed in the HFS group, followed by the THA group and the control group. Lischke and colleagues have indicated RMSSD as a marker for an immediate reaction to stress, which is in line with our findings [28]. The results of the operated groups showed that the lower the values of RMSSD, the worse the recovery ability of a person, and the worse the HRV. This can perhaps be attributed to a more inactive lifestyle of those in the operated groups, which is in line with lower results in CC, BMI and grip strength in these groups. Contrary to our expectations, results of the THA group were similar at the two MTPs. This could also be a result of an inactive lifestyle, possibly caused by pain, being overweight or perhaps the level of difficulty of the task, as postulated by Tegegne et al. [29]. Additionally, the connection between blood pressure and HRV was emphasized with our findings of higher blood pressure related to lower levels of HRV.

*SD1* results will not be further expanded upon here, due to their redundancy with RMSSD, since identical values were observed, as has also been observed in other research from Shaffer and Ginsberg [30].

### Limitations

Apart from the general limitations of non-invasive hemodynamic monitoring in elderly patients, the small sample size and the observational nature of the study should be considered as limitations of this study. A large number of participants in the HFS group were excluded or dropped out, due to further pathological issues and also due to not being able to sit quietly during ECG recording. Thus, patients in the HFS group do not paint a representative picture of the traumatic ward, which could be attributed to a selection bias. In further studies, a larger number of participants should be included, possibly with different entrance criteria, such as an open upward age limit or additional ECG recording options, such as preoperative or perioperative ECG reports or data records at time of admission. Further, we conducted our experiment with patients from one single hospital in Austria, who were being medically treated for hip fracture or for a total hip arthroplasty, and compared these patients to healthy adults. These data cannot be generalized to the overall population of patients who are subject to HFS or THA.

## Conclusions

Two groups of patients with hip surgery, HFS and THA, and a control group were investigated in this study, in order to examine differences in anthropometric and cardiovascular parameters. For anthropometric parameters, the most crucial findings were that the control group showed significantly higher CC than the HFS group, and significantly higher grip strength than both the HFS and THA groups, which may be indicative of a less active lifestyle in patients with hip surgery. Interestingly, differences in cardiovascular parameters (HR, RMSSD) were only found between HFS patients and the control group, but not between the hip patient groups. While this may indicate greater similarities between HFS and THA patients than has been previously assumed, further investigations are required to ascertain whether these findings can be applied to a larger cohort of hip surgery patients.

## Data Availability

The dataset used and/or analysed during the current study are available from the corresponding author on reasonable request.

## Abbreviations

ANOVA: Analysis of variance
BMI: Body mass index
CC: Calf circumference
DBP: Diastolic blood pressure
ECG: Electrocardiogram
EDF: European data format
FD: Frequency domain
HF: High frequency
HFS: Hip fracture surgery
HR: Heartrate
HRV: Heart rate variability
Hz: Hertz
ISAK: International Society for the Advancement of Kinanthropometry
LF: Low frequency
ln: Natural logarithm
M: Mean
ms: Milliseconds
MTP: Measurement time point
NN: Normal to normal R–R intervals
OECD: Organization for Economic Co-Operation and Development
RMSSD: Root mean square of successive heartbeat interval differences
SBP: Systolic blood pressure
SD: Standard deviation
SD1: Standard deviation of the short-term NN interval variability
SD2: Standard deviation of the long-term NN interval variability
SDNN: Standard deviation of normal to normal R-R intervals
SPSS: Statistical Packages for the Social Sciences
TD: Time domain
THA: Total hip arthroplasty
